# Credibility Assessment of a Subject-Specific Mathematical Model of Blood Volume Kinetics for Prediction of Physiological Response to Hemorrhagic Shock and Fluid Resuscitation

**DOI:** 10.3389/fphys.2021.705222

**Published:** 2021-09-16

**Authors:** Bahram Parvinian, Ramin Bighamian, Christopher George Scully, Jin-Oh Hahn, Pras Pathmanathan

**Affiliations:** ^1^Department of Mechanical Engineering, University of Maryland College Park, College Park, MD, United States; ^2^Office of Science and Engineering Laboratories, Food and Drug Administration, Silver Spring, MD, United States

**Keywords:** subject-specific, model credibility assessment, workflow, model validation, fluid resuscitation, mathematical model

## Abstract

Subject-specific mathematical models for prediction of physiological parameters such as blood volume, cardiac output, and blood pressure in response to hemorrhage have been developed. *In silico* studies using these models may provide an effective tool to generate pre-clinical safety evidence for medical devices and help reduce the size and scope of animal studies that are performed prior to initiation of human trials. To achieve such a goal, the credibility of the mathematical model must be established for the purpose of pre-clinical *in silico* testing. In this work, the credibility of a subject-specific mathematical model of blood volume kinetics intended to predict blood volume response to hemorrhage and fluid resuscitation during fluid therapy was evaluated. A workflow was used in which: (i) the foundational properties of the mathematical model such as structural identifiability were evaluated; (ii) practical identifiability was evaluated both pre- and post-calibration, with the pre-calibration results used to determine an optimal splitting of experimental data into calibration and validation datasets; (iii) uncertainty in model parameters and the experimental uncertainty were quantified for each subject; and (iv) the uncertainty was propagated through the blood volume kinetics model and its predictive capability was evaluated *via* validation tests. The mathematical model was found to be structurally identifiable. Pre-calibration identifiability analysis led to splitting the 180 min of time series data per subject into 50 and 130 min calibration and validation windows, respectively. The average root mean squared error of the mathematical model was 12.6% using the calibration window of (0 min, 50 min). Practical identifiability was established post-calibration after fixing one of the parameters to a nominal value. In the validation tests, 82 and 75% of the subject-specific mathematical models were able to correctly predict blood volume response when predictive capability was evaluated at 180 min and at the time when amount of infused fluid equals fluid loss.

## Introduction

Subject-specific mathematical models may be regarded as mathematical models that can be adapted to each subject to provide individualized predictions. Because such mathematical models are tailored to each subject, they have the potential to enable precision medicine particularly when they are utilized in decision support systems applications where the therapy decision recommendations need to be optimized based on subject-specific physiology, as opposed to a population of subjects (Neal and Bassingthwaighte, 2007; Marquis et al., 2018). Additional applications of subject-specific mathematical models beyond decision support include the design and evaluation of autonomous therapy controllers (Bighamian et al., 2016a, 2018; Mirinejad et al., 2019; Parvinian et al., 2019).

Subject-specific mathematical models can perform their predictive purpose in various capacities. They have the potential to augment clinical trials either by reducing the size of the trial or to inform and optimize trial design (Faris and Shuren, 2017). A precursor to clinical applications of subject-specific mathematical models is their use in pre-clinical safety evidence generation where they can be used to build a cohort of *in silico* subjects to complement and/or replace traditional animal studies (Kovatchev et al., 2009; Jean-Quartier et al., 2018; Mirinejad et al., 2019; Viceconti et al., 2020). They further enable rapid prototyping and evaluation of new system designs potentially without having to reperform animal studies. This method of pre-clinical safety evidence generation is particularly helpful when significant inter- or intra-subject variability is expected but is difficult to capture in animal studies due to sample size requirements leading to financial or ethical limitations.

The vision of replacing animal studies with *in silico* studies can only be realized if the credibility of the mathematical models is established for the particular purpose they are intended to serve. Credibility is defined as the trust, based on all available evidence, in the predictive capability of the mathematical model (ASME, 2018). Credibility assessment involves numerous activities including model verification (confirming a computational model is an accurate implementation of an underlying mathematical model), model validation (comparison of mathematical model’s predictions against real-world data), applicability assessment (evaluating the relevance of validation activities for the intended use of the mathematical model), as well as related activities pertaining to model parameters including identifiability analysis (for parameters calibrated to experimental data, determining if the data is sufficient to constrain the parameters) and Uncertainty Quantification (UQ) (determining the uncertainty in model parameters and the resultant uncertainty in the outputs of the mathematical model). Major challenges with demonstrating credibility of subject-specific mathematical models include, but are not limited to (1) ensuring the soundness of model structure *a priori*, (2) reliably tuning model parameters to each specific subject, a process known as model calibration, given limitations in quantity and quality of experimental data, and (3) quantifying uncertainty of mathematical model’s prediction and experimental uncertainty to evaluate the mathematical model’s predictive capability. Methods for overcoming these challenges often resort to *ad hoc* and iterative mathematical model development and evaluation processes which can be inefficient. Therefore, it is desirable to utilize a systematic workflow when evaluating subject-specific mathematical models in the application area of interest. In this paper, we define and apply such a workflow. The workflow focuses on activities related to parameter identifiability, UQ, and model validation; other credibility assessment activities such as verification and applicability analysis are out of scope. As such, this work includes a partial credibility assessment focused on model validation and UQ.

One promising application of subject-specific mathematical models focuses on fluid resuscitation therapy in response to hemorrhage and fluid infusion, which has been investigated extensively using animal models (Warner and Hahn, 2010; Navarro et al., 2015; Norberg et al., 2016). However, there are still open and important questions that need to be answered to determine optimal fluid delivery to subjects in the case of hemorrhage, especially in regard to the type, amount, and timing of fluid delivery to be tailored to specific subjects (Navarro et al., 2015). Several studies have leveraged the utility of subject-specific mathematical models to predict physiological responses such as change in blood volume (BV) or mean arterial pressure (MAP) in response to fluid delivery (Bighamian et al., 2017, 2018; Tivay et al., 2020). Furthermore, studies have been conducted to use these mathematical models to guide autonomous therapy of fluids (Jin et al., 2019). However, the ability to reduce or replace animal studies hinges on the credibility of the proposed mathematical models whose subject-specific predictions (e.g., BV response to hemorrhage and fluid infusion) are utilized. Such mathematical models are often demonstrated to be able to fit the previously generated data used for parameter calibration, but in general evidence for their overall predictive capability is limited (Parvinian et al., 2019). In particular, it is important that subject specific mathematical models are shown to be able to predict physiological behavior beyond the information they are built upon (Ljung, 2008; Batzel et al., 2013).

In this work, we define a step-by-step assessment workflow for evaluating subject-specific mathematical models calibrated to time-series data and apply it to investigate the credibility of a previously introduced subject-specific BV kinetics model (Bighamian et al., 2018). The BV kinetics model can be used for the prediction of BV response to fluid infusion after episodes of controlled hemorrhage in an ovine model. Table 1 overviews the steps in our workflow with corresponding information obtained from each step. The first step is to define the model purpose and the question that the mathematical model should answer. The second step is to evaluate the identifiability of parameters *a priori* (i.e., global structural identifiability), that is, whether the subject-specific parameters calibrated to data from each subject can theoretically be uniquely determined given the structure of the mathematical model assuming the most informative and noise-free data. The third step involves evaluating the quality of data to be used for calibration to prospectively determine an optimal calibration window. This step will determine how to split each subject’s experimental data into calibration data and validation data, such that the calibration data provides sufficient information to determine subject-specific parameters but there remains adequate data for testing the mathematical models’ predictive capability using the validation data. The fourth step is to proceed with model calibration and optimize the parameters of the mathematical model for each specific subject. The fifth step involves performing the post-calibration identifiability analysis which enables UQ, in which the uncertainty in the calibrated parameters is estimated. As the penultimate step, we perform UQ on the experimental data to enable the comparison of the mathematical model predictions considering the propagated model uncertainty with the experimental data that were not used in calibration—a test of the predictive capability. For the BV kinetics model this step is conducted at two physiologically relevant time points in the course of fluid therapy: at the conclusion of fluid therapy, and when the amount of fluid infused equals the volume of fluid loss.

**TABLE 1:** Step by step workflow followed for subject specific model reliability assessment.

Steps	Process step	Question addressed by each step
1	Define the role of the model	How will the model be used?
2	Global Structural Identifiability Analysis	Can the parameters of the model theoretically be uniquely identified *a priori* using noise-free and experimental data?
3	Pre-calibration Identifiability Analysis	• Is the data set informative to allow for identifiable parameters? • Can the time series data for each animal set be split to allow for independent validation? If so, what is the optimal calibration time window?
4	Model Calibration	• Is the model able to match the experimental data when parameters are calibrated? • Do the calibrated parameters take physiologically reasonable values? • Are the confidence intervals of the parameters within a physiologically plausible region?
5	Post calibration identifiability analysis and uncertainty quantification	• Is the uncertainty in the identified parameters bounded? • What is the confidence interval of the identified parameters? • Does the confidence interval of the parameters include values that are physiologically plausible?
6	Experimental Data Uncertainty Quantification	What is the uncertainty in the experimental data to be compared with model predictions?
7	Quantitative Validation and Predictive Capability Assessment	What is the predictive capability of the model considering its purpose accounting for model and experimental uncertainty?

Overall, we introduce a cohesive process that connects various steps of credibility assessment associated with the BV kinetics model that are often scattered in previous published work. For example, while UQ has been conducted for some physiological models (Marquis et al., 2018), the non-trivial question of structural identifiability has not been addressed in previous studies of BV kinetics models. Furthermore, while the performance of BV kinetics models has been evaluated extensively using various measures of calibration fit (Parvinian et al., 2019), to the best of our knowledge, such performance has not been assessed on experimental data independent of the parameter calibration process.

## Materials and Methods

### Blood Volume Kinetics in Hemorrhage and Fluid Resuscitation

In this work, we aim to evaluate the credibility of a 3-parameter mathematical model of BV kinetics in response to hemorrhagic blood loss and fluid infusion to serve as a case study (Equation 1):


(1) $$△\,{\ddot{V}}_{B}\,\left(t\right)+K_{p}\,△\,{\dot{V}}_{B}\,\left(t\right)=\left[\dot{U}\,\left(t\right)-\dot{V}\,\left(t\right)\right]+\frac{K_{p}}{1+\alpha_{u}}\,U\,\left(t\right)-\frac{K_{p}}{1+\alpha_{v}}\,V\,\left(t\right)$$

where △*V*_*B*_ is the change in BV, *U*(*t*) and *V*(*t*) are fluid infusion and hemorrhage rates, respectively, and the parameters α_*u*_,α_*v*_,*K*_*p*_ are the ratio of volume gain between intravascular and interstitial compartments, the ratio of volume loss between intravascular and interstitial compartments, and the rate of fluid shift between intravascular and interstitial compartments, respectively. This mathematical model was previously published in 3-parameter and 4 parameter variants (Bighamian et al., 2016b, 2018). In this work, we considered the 3-parameter BV kinetics model. Furthermore, since the rate of urine output was negligible compared to the rates of hemorrhage and fluid infusion, it was not included as a model input for data fitting.

### Animal Study Protocol

**FIGURE 1:**
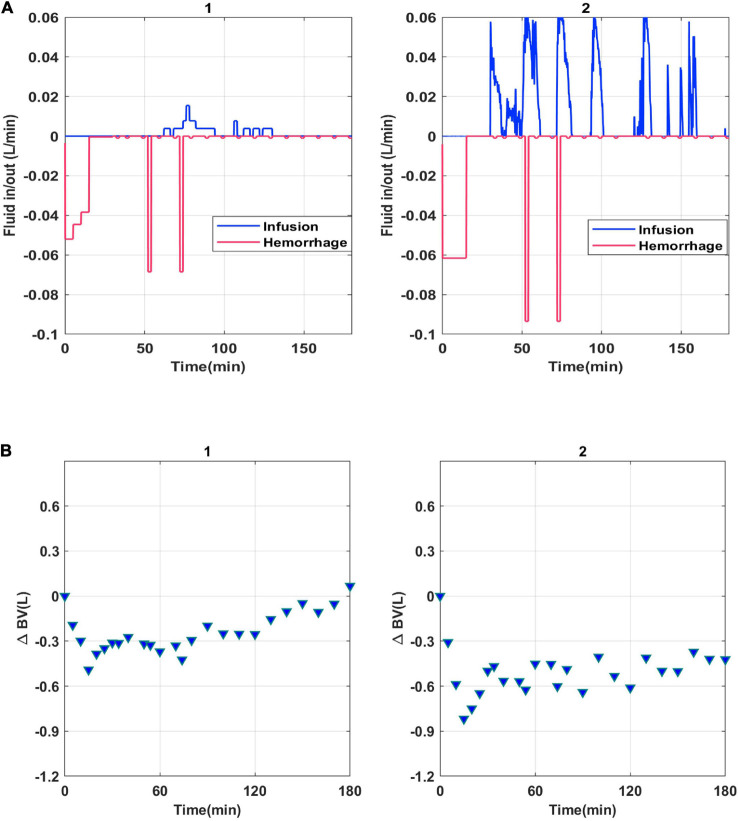
Model fluid input **(A)** and change in blood volume **(B)** for subjects 1 and 2.

The experimental data used in this work were collected retrospectively from 22 conscious sheep undergoing intravenous blood loss and fluid infusion. The measurements included the rates of hemorrhage and fluid infusion, urine output, and BV (Figure 1). The data collection protocol was approved by the Institutional Animal Care and Use Committee (IACUC) at the University of Texas Medical Branch and is described in detail elsewhere (Rafie et al., 2004). All 22 animals received lactated Ringers (LR) solution. The duration of study for each animal was 180 min. After the baseline data were recorded, an initial hemorrhage (25 mL/kg) was performed over 15 min. Fluid infusion was started 15 min after the end of the hemorrhage and continued for 150 min. Second and third hemorrhage (5 mL/kg) were performed 50 and 70 min after the start of the initial hemorrhage, each of which lasted for 5 min. While hemorrhage protocol remained constant across the subjects, fluid infusion was varied based on predetermined rates and algorithms as described in Rafie et al. (2004). In each animal, baseline BV was measured *via* indocyanine green dye (ICG). Hematocrit, the ratio between the red blood cell volume (RBCV) and BV, was measured before and throughout the experiment at 5–10 min intervals and was used to measure the fractional change in BV.

### Credibility Analysis of Mathematical Model

As the first step, it is essential to prospectively define the role of the mathematical model and the question it will be used to address. In this work we have proposed to use the BV kinetics model as a decision support tool to predict BV response to hemorrhage and fluid infusion in animal subjects.

#### Pre-calibration Identifiability Analysis

##### Structural Identifiability Analysis

Structural identifiability analysis focuses on the soundness of model structure independent of data quality. The goal of structural identifiability analysis is to prove that it is theoretically possible to identify unique model parameter values given the planned model calibration data, assuming the data is noise free. In this work, we leverage the Laplace transform approach (Pronzalo and Walter, 1997) by virtue of the linearity of the BV kinetics model and the relatively small number of parameters involved therein.

##### Test of Data Quality Prior to Calibration

We used the singular value decomposition (SVD) to evaluate the quality of the data to be used for calibration (Keesman, 2011). The first step allowing for SVD to be leveraged was to convert the mathematical model to linear regression form as in Equation 2:


(2) △⁢V¨B⁢(t)-[U.⁢(t)-V.⁢(t)]=[Kp1+αu⁢Kp 1+αv⁢Kp]⁢[U⁢(t)-V⁢(t)-△⁢V.B⁢(t)],t=1,⋯,N

For each subject, the regressor matrix is a matrix with rows corresponding to experimental measurements for *U*(*t*), *V*(*t*) and $△\,{\dot{V}}_{B}\,\left(t\right)$at N distinct time instants, respectively. SVD was applied to the regressor matrix for each subject to compute the three singular values of the regressor matrix and the corresponding principal directions as determined by the right singular vector. If the SVD results demonstrate small singular values in the direction of certain parameter(s) relative to the others (i.e., the regressor matrix is not of full rank and the estimation problem is ill conditioned), consistently across all animals, this indicates unidentifiability or low identifiability of the associated parameter(s). Those parameters can then be set to physiologically plausible constant values. In this case, we would reapply the SVD to the new mathematical model in order to evaluate the data quality for calibration of the remaining parameters.

##### Selection of Calibration Window

Next, the experimental data was split into calibration and validation datasets based on the results of SVD analysis of the regressor matrix. Specifically, a time *T*_*c*_ was identified based on the magnitude of the singular values and their associated principal direction as determined by the right singular vector, and then data for the time interval [0 min, *T*_*c*_] was used for calibration, with the remaining experimental data [*T*_*c*_, 180 min] saved for model validation. It is important to select *T*_*c*_ with consideration of the overall experimental protocol to enable independent model evaluation under inputs and conditions that were not included to the calibration process.

#### Model Calibration

Next, model calibration was performed, using only the data in the range [0 min, *T*_*c*_ min] for each animal. The Levenberg–Marquardt algorithm was used to solve the non-linear least squares optimization problem in Equation 3 with a proportional error model to account for the uncertainty associated with the BV measurements (Aladangady et al., 2008):


(3) $${\text{P}}^{*}=\left\{\alpha_{u}^{*},\alpha_{v}^{*},K_{p}^{*}\right\}=\arg\,\min_{\text{P}}\,{||\left(\frac{\Delta \,{\text{V}}_{B,m}\,\left(P,t\right)-\Delta \,{\text{V}}_{B,e}\,\left(t\right)}{\left|\Delta \,V_{B,m}\,\left(P,t\right)\right|}\right)||}_{2}$$

where △V_B,m_(P, t)is the model predicted change in BV at time *t* using parameters *P*, and △V_*B*,*e*_(*t*) is the experimentally measured change in BV at time *t*. The quality of fit was evaluated using the root-mean-squared normalized error (RMSNE) as defined by:


(4) $$\text{RMSNE}={\left(|\bar{△{\text{V}}_{B,e}|}\right)}^{-1}\sqrt{\frac{\sum_{i=1}^{N}{\left(△\,{\text{V}}_{B,m}\,\left(t_{i}\right)-△\,{\text{V}}_{B,e}\,\left(t_{i}\right)\right)}^{2}}{N}}$$

Information that calibration results provide regarding model credibility is limited. Successful calibration indicates that the structure of the mathematical model fits the data. Calibration does not provide information about generalizability or predictive power for conditions other than those included in the calibration dataset.

#### Post-calibration Identifiability Analysis and Parameter Uncertainty Quantification

Identifiability of the parameters should be evaluated after parameters have been estimated. The practical identifiability and data quality tests in the pre-calibration step proposed in section “Pre-calibration Identifiability Analysis”. serve as a necessary but not sufficient condition for demonstrating parameter identifiability. SVD only provides information regarding the output sensitivity of subject-specific parameters but does not discern relative from absolute identifiability.

In this step, we first visualized the cost function (see Equation 3) contours in parameter space for each subject, and then calculated the asymptotic 95% confidence region of the calibrated parameters using the parameter covariance matrix computed during the calibration stage (Pronzalo and Walter, 1997). This enables visual determination of correlation between parameters and the potential associated impact on parameter identifiability. If the contour region is unbounded in the direction of one particular parameter, resulting in an unbounded confidence region, this indicates that that parameter was practically unidentifiable (Raue et al., 2009), in which case we fix it to a physiologically reasonable value and repeat everything from Step 4.

We accounted for the uncertainty in the calibrated model parameters by assuming the calibrated parameters for any subject were multivariate normally distributed with covariance matrix as calculated in Step 4. We propagated this uncertainty through the mathematical model when running the validation simulations (Monte Carlo with 10,000 samples). In subjects 2, 10, and 14 extremes of confidence regions resulted in physiologically implausible *K_p_* values (e.g., *K_p_* = 0), These samples consisted of 1.1, 1.7, and 3.5% of the total samples in subject 2, 10, and 14, respectively, and were excluded from further analysis.

#### Experimental Uncertainty Quantification

In order for the validation results to be considered meaningful, experimental uncertainty must be quantified and compared with computational uncertainty. Uncertainty in the change in BV measurements may be quantified by evaluating the standard deviation of the baseline BV (BV_0_) which is based on ICG measurement of plasma volume (PV_0_) and hematocrit (Hct_0_). Since the animal study was not designed to have repeated measurements of BV at multiple points during the course of the experiment, the standard deviation of such measurements cannot be determined based on the available data. While accuracy of BV measurements for the ICG technique has been studied against gold standards such as radiolabeled albumin technique (Aladangady et al., 2008), published literature on repeatability or reproducibility of BV measurement is scarce (Imai et al., 2000) and few studies that have attempted it have focused on the specific BV measurement technique used in our experimental study. However, it is possible to extract BV measurement standard deviation and quantify its proportionality to changes in BV from published literature on hemodialysis studies (Aladangady et al., 2008). Experimental uncertainty quantified based on this reference, which utilized a method validated using the ICG technique, yielded a proportionality constant of 0.2 between measurements of change in BV and their standard deviation.

#### Model Validation

The above steps ensure that the parameters of the mathematical model are reliable, and that their uncertainty is adequately quantified, and propagated for quantitative comparison with experimental uncertainty. We only then proceeded to assess the predictive capability of the mathematical model assuming the ultimate goal of the mathematical model is to provide subject-specific prediction of changes to BV response to hemorrhage and fluid resuscitation to support pre-clinical safety assessment. Given this context of use, two validation tests were formulated: (i) assessment of prediction of responsiveness to fluid therapy at the conclusion of the study (i.e., 180 min), in which the general responsiveness of BV was defined by the rise of BV above normovolumia (△V_*B*,*e*_ = 0) in response to subject-specific fluid therapy (i.e., irrespective of variability in fluid therapy); and (ii) assessment of prediction of responsiveness to fluid therapy defined by BV restoration to normovolemic state at subject-specific time instant in response to cumulative fluid infusion equal to cumulative hemorrhage. For each validation test, we evaluated the probability that the mathematical model’s binary classification of responsiveness/non-responsiveness was in agreement with that observed experimentally.

## Results

### Experimental Subject-Specific Results

Subject-specific experimental results are shown in Figure 1 for two animal subjects. Subject-specific hemorrhage and infusion protocols are shown in Figure 1A. Each subject received the same volume of hemorrhage normalized by subject weight. However, the input infusion profile delivered varied across subjects depending on pre-determined experimental protocol (Rafie et al., 2004). Figure 1B depicts the experimental measurement of change in BV response to hemorrhage and infusion shown in Figure 1A.

### Pre-calibration Identifiability Analysis

#### Structural Identifiability

It can be shown using the Laplace transform method that the mathematical model is globally structurally identifiable. The proof is provided in Supplementary Material 1.

#### Pre-calibration Practical Identifiability Analysis and Calibration Window

**FIGURE 2:**
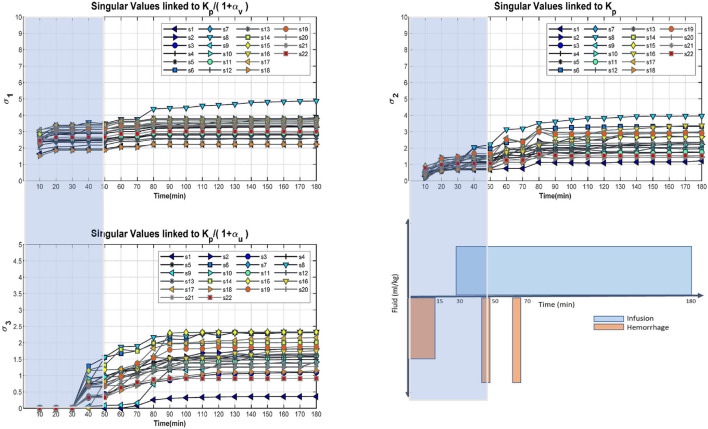
Entire cohort singular values for Kp 1+αv, *K_p_*, and Kp 1+αu as determined by the approximation of the right singular matrix. Plot on the bottom right indicates the experimental protocol used for data collection depicting timing of fluid hemorrhage and infusion. X-axis of the singular value plots relates to the amount of experimental data under consideration, for example *T*_*c*_ = 50 min corresponds to considering all data between 0 and 50 min. The calibration window of 0–50 min was selected because it was the largest time window that would allow for evaluation of model prediction in an independent data segment (i.e., 50–180 min) containing both infusion and hemorrhage while still having relatively large singular values for majority of subjects in the study. Overall, it can be concluded that α_*v*_ is more identifiable than α_*u*_.

The main purpose of this step is to identify a calibration window to allow for independent validation of the model and second and to gain qualitative insight on relative order of identifiability of the parameters. Results of the SVD analysis are provided in Figure 2. After approximation of the right singular vectors, the singular values’ principal directions were generally aligned with the axes in parameter space; the first (largest) singular value corresponded to Kp 1+αv, the second to *K*_*p*_, and the third to Kp 1+αu. The x-axis of the plots represents the amount of experimental data used in constructing the regressor matrix: *t* = 180 min means that the regressor matrix used all the experimental data, *t* = 50 min means that the regressor matrix used only the data up to 50 min. Therefore, increasing data is used as t increases, which in turn increases the singular values. The results reveal that generally the order of identifiability of parameter isKp 1+αv>Kp>Kp 1+αu which indicates that α_*v*_ is better estimable (i.e., with more accuracy) than α_*u*_ and identifiability of *K_p_* cannot be directly compared with other parameters (see the “Discussion” section for details). This is not to say which parameters are identifiable but rather their relative identifiability. Based on these results, we chose *T*_*C*_ = 50 min, that is, the calibration window as 0–50 min, because this was the smallest time window containing both hemorrhage and fluid infusion and having relatively large singular values for the majority of subjects in the study. The motivation for choosing the smallest possible time window for calibration is to leave the largest possible time window (50–180 min) for model validation. An alternative choice of calibration window of 0–80 min could have been made based on the fact that singular values do not increase substantially after 80 min. However, with this choice there would be no hemorrhage in the validation window (see Figure 1).

### Calibration and Post-Calibration Practical Identifiability Analysis

**FIGURE 3:**
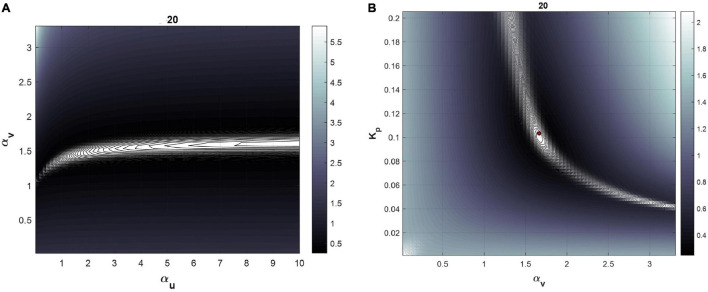
Cost function visualization for the subject 20 for the three parameter model. **(A)** Cost function as a function of α_*u*_ and α_*v*_at fixed *K*_*p*_. The direction of unbounded ellipses representing cost function contours demonstrates unidentifiability of α_*u*_. **(B)** Cost function as a function of *K*_*p*_ and α_*v*_ at fixed α_*u*_ values.

**FIGURE 4:**
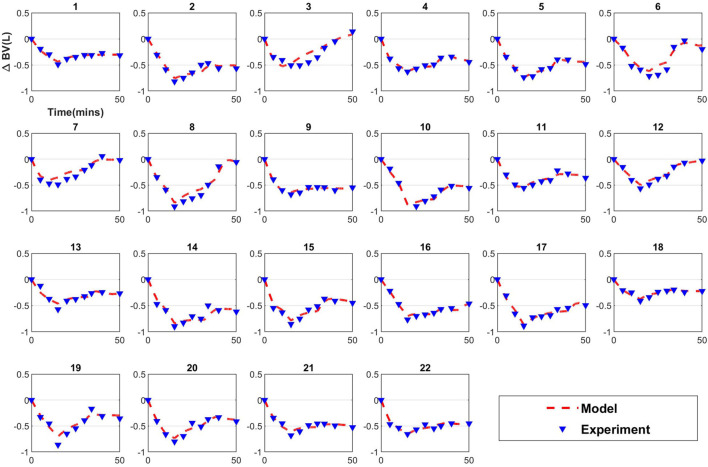
Calibration results for the two parameter model.

Table 2 lists the values of the identified parameters after calibration to the 0–50 min data, and the corresponding RMSNE. For all subjects except subjects 3, 13, and 16, α_*u*_ was not identifiable. This was confirmed by visualizing the cost function contours in the neighborhood of the identified parameters demonstrating an unbounded contour in the direction of α_*u*_ (see Figure 3A for example with subject 20) and bounded regions containing identified values of α_*v*_ and *K*_*p*_ (see Figure 3B). For these subjects, the error function continues to decrease as α_*u*_ increases and no global minimum exists. For subjects 3, 13, and 16, a finite value of α_*u*_ was found but α_*v*_ was found to be unidentifiable or *K_p_* took values significantly beyond cohort average. Since α_*u*_ was unidentifiable for nearly all subjects, it was set to 3 was calculated based on average value of α_*u*_ identified from four Ringer’s infusion protocols reported in previous studies (Bighamian et al., 2016b). The calibration process was then repeated for the two-parameter model; results are provided in Table 3. With the two-parameter model, calibrated α_*v*_ and *K_p_* within reasonable physiological ranges and with physiological relevance were found for all subjects except subjects 3 and 16. These subjects were considered subjects for which the mathematical model fails (see section “Discussion”) and were excluded from further analysis. The fitted mathematical models closely matched the experimental data, as seen in Figure 4 (see RMSNE column in Table 2 for quantitative measure of fit).

**TABLE 2:** Calibration Results for three parameter model.

Subject #	α_u_ [.]	α_v_[.]	K_p_ min^–1^	RMNSE%
1	inf	1.39	0.14	9.74
2	inf	0.53	0.11	11.26
3	−0.43	Inf	0.04	24.59
4	inf	0.86	0.13	3.93
5	inf	0.92	0.10	4.87
6	inf	2.40	0.11	25.14
7	inf	4.69	0.12	29.91
8	inf	1.71	0.07	14.55
9	inf	0.69	0.18	6.20
10	inf	0.50	0.06	10.21
11	inf	1.89	0.10	11.52
12	inf	3.14	0.06	12.93
13	0.30	1.11	0.67	22.65
14	inf	0.41	0.15	11.55
15	inf	1.08	0.11	10.00
16	2.32	0.57	0.70	5.42
17	inf	1.11	0.11	7.31
18	inf	1.97	0.17	12.15
19	inf	2.21	0.08	17.75
20	inf	1.66	0.10	9.82
21	inf	1.09	0.25	9.21
22	inf	0.96	0.12	7.33

*Inf, infinity; that is, the solver failed to converge for this parameter. α_*u*_ was found to be unidentifiable for all parameters except for subject 3, 13, and 16. However, other parameters in these subjects were calibrated to values beyond physiological range.*

**TABLE 3:** Uncertainty Quantification of the parameters for two parameter model after fixing α**_u_** to 3.

Subject#	α_u_[.]	α_v_[.]	Kp min^–1^	RMNSE%	CIα_v_ low	CIα_v_ high	CI Kp low	CI Kp high
1	3.00	1.40	0.14	9.73	1.05	1.75	0.07	0.22
2	3.00	0.47	0.13	12.42	0.31	0.64	0.00	0.27
3	3.00	inf	0.06	33.93	inf	inf	0.01	0.11
4	3.00	0.75	0.16	5.50	0.68	0.82	0.12	0.21
5	3.00	0.81	0.13	6.00	0.71	0.91	0.08	0.17
6	3.00	1.81	0.13	25.37	1.30	2.32	0.03	0.23
7	3.00	3.20	0.16	30.78	1.52	4.89	0.00	0.32
8	3.00	1.35	0.09	15.87	1.11	1.59	0.06	0.13
9	3.00	0.68	0.19	6.27	0.56	0.81	0.07	0.31
10	3.00	0.43	0.07	10.61	0.23	0.62	0.00	0.15
11	3.00	1.76	0.11	11.71	1.17	2.35	0.05	0.18
12	3.00	2.29	0.07	15.91	2.15	2.44	0.06	0.09
13	3.00	1.44	0.18	18.41	0.99	1.90	0.02	0.34
14	3.00	0.35	0.21	13.13	0.19	0.51	0.00	0.47
15	3.00	0.94	0.12	11.31	0.75	1.14	0.06	0.19
16	3.00	0.59	0.59	5.49	0.53	0.65	0.30	0.88
17	3.00	1.05	0.12	7.80	0.81	1.30	0.06	0.18
18	3.00	1.85	0.19	12.47	1.50	2.21	0.10	0.27
19	3.00	2.10	0.10	19.12	1.04	3.16	0.02	0.17
20	3.00	1.51	0.11	10.57	1.22	1.80	0.07	0.16
21	3.00	1.03	0.27	9.86	0.86	1.21	0.10	0.44
22	3.00	0.91	0.13	7.12	0.73	1.10	0.06	0.20

*The high and low values represent 95% confidence intervals for the calibrated parameters.*

*Subject 3 and 16 continued to have parameter values beyond reasonable physiological range.*

### Parameter Uncertainty Quantification

**FIGURE 5:**
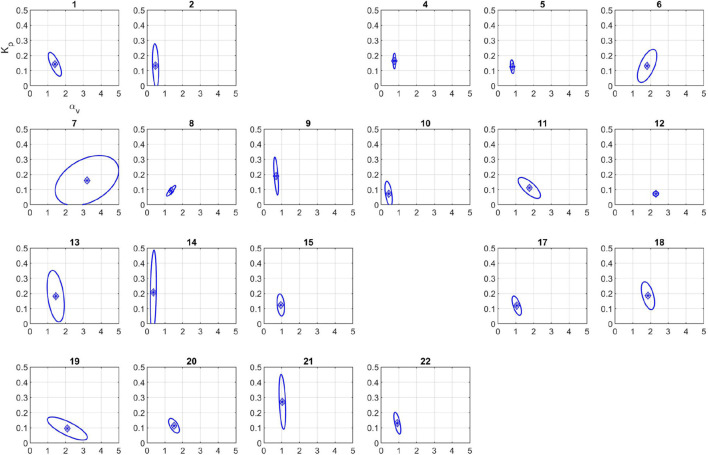
95% confidence regions for the calibrated parameters using the two parameter model. Subjects 3 and 16 were excluded because calibrated parameters were outside physiological range (see section “Discussion”).

The 95% confidence regions for the calibrated model parameters are plotted in Figure 5. The corresponding 95% confidence intervals for each model parameter are listed in Table 3. The uncertainty in each subject’s identified parameter values varies considerably between the subjects; it is very small for many animals, but it is considerable for others (e.g., subject 7).

### Uncertainty Propagation and Model Validation

**FIGURE 6:**
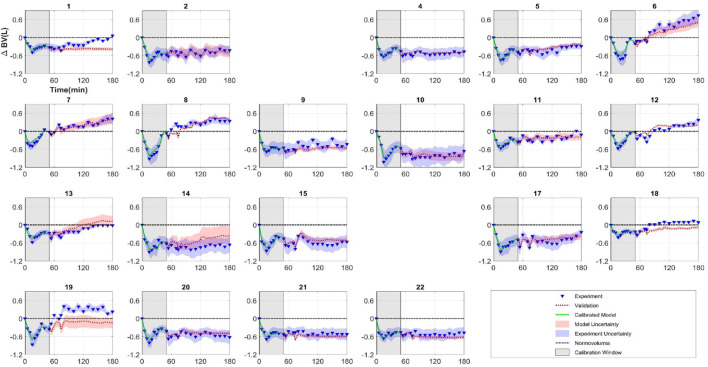
Subject-specific model calibration and validation. Each figure represents a different animal (subjects 3 and 16 were excluded because parameters were not identifiable for these subjects). Model was calibrated to experimental data between 0 and 50 min (model: green line, experiment: triangles). Model was then simulated from 50 to 180 min (red dashed line) and can be compared against experimental measurements (triangles) (model validation). Shaded regions represent 95% confidence intervals.

Figure 6 plot the results of each subject’s calibrated model for the entire 180 min (see red dashed line), together with the corresponding experimental data (triangles). The first 50 min corresponds to model calibration (the same data as Figure 4), whereas the 50–180 min results represent model validation. The parameter’s quantified uncertainty discussed in section “Parameter Uncertainty Quantification” was also propagated through the mathematical model using Monte Carlo sampling (*N* = 10,000) to derive the uncertainty in the model predictions. These are represented in Figure 6 as a 95% confidence interval in model output (red shaded region) and can be compared to the 95% confidence interval for the experimental measurements (blue shaded region) which is based on the values discussed in section “Model Validation”.

Finally, the results of the binary tests to evaluate the mathematical model’s prediction of fluid responsiveness for critical time points during therapy while accounting for the uncertainty on both the prediction and the experiment are presented in Table 4.

**TABLE 4:** Results of quantitative validation tests.

Subject	P_*agreement,180*_	T* (min)	P_agreement,T_* -
1	0.00	–	n/a
2	1.00	96	1.00
4	1.00	–	n/a
5	1.00	108	1.00
6	1.00	78	1.00
7	1.00	84	1.00
8	1.00	72	0.02
9	1.00	–	n/a
10	1.00	–	n/a
11	1.00	180	1.00
12	1.00	90	1.00
13	0.04	102	1.00
14	0.98	84	1.00
15	1.00	–	n/a
17	1.00	174	1.00
18	0.00	174	0.00
19	0.04	177	0.04
20	1.00	–	n/a
21	1.00	–	n/a
22	1.00	–	n/a

P_*agreement,180*_ is the probability of the model and experiment both being greater than 0, or both being less than 0, at *t* = 180 min. T* is the time at which cumulative infusion was equal to cumulative hemorrhage (no such time exists for some subjects). P_agreement,T_* is the probability of the model and experiment both being greater than 0, or both being less than 0, at *t* = T*.

## Discussion

In this work, we have evaluated the credibility of a subject-specific mathematical model of BV changes in response to hemorrhage and fluid infusion, using a workflow that quantitatively and systematically evaluates different aspects of the mathematical model, including structural identifiability, calibration, and post calibration parameter uncertainty. While it is quite common for parameterized mathematical models to first be evaluated qualitatively, our proposed process recommends specific pre- and post-calibration steps for quantitative credibility assessment of a mathematical model in a cohesive and unified workflow. The need for this workflow arises from the importance of the order of the steps taken toward establishing the credibility of a mathematical model to avoid problems such as parameter unidentifiability or absence of a validation window with sufficient information to allow predictive capability evaluation in the latter steps of model evaluation process. Both issues are commonly faced in subject-specific modeling, as the experimental data used for calibration are typically collected retrospectively and not obtained from the protocols that were intended to inform a model validation and credibility assessment.

As the first step toward evaluating the credibility of a subject-specific mathematical model, the fundamental soundness of model structure and whether it allows for unique parameter identification should be assessed. This is because internal structural inconsistencies may result in unidentifiable parameters regardless of the quality of the data used for model calibration. The test for structural soundness is known as structural or theoretical identifiability (Pronzalo and Walter, 1997; Raue et al., 2009). A model is structurally identifiable if for almost any $\tilde{\text{P}}$:


(5) $$ℳ\,\left(\text{P}\right)=ℳ\,\left(\tilde{\text{P}}\right)\Rightarrow P=\tilde{\text{P}}$$

where ℳ denotes model structure, P and $\tilde{\text{P}}$ represent model parameters. Under the assumptions that (i) the model structure characterizes the modeled process faithfully and (ii) the experimental data are rich, informative, and noise free, this test asks whether model parameters calibrated with the data would be unique (Pronzalo and Walter, 1997; Raue et al., 2009).

Global structural identifiability can be assessed using a variety of methods including the Laplace transform, similarity transformation, Taylor series, and state isomorphism approaches (Chis et al., 2011). Numerical approaches can also be leveraged for preliminary tests of local identifiability (Pronzalo and Walter, 1997). The choice of the method to demonstrate structural identifiability largely depends on the model structure (i.e., linear-in-variables or parameters) and complexity. Each method presents unique advantages and limitations. For example, the Laplace transform approach leveraged in this work is suitable for linear-in-variable models with small numbers of parameters and is the least computationally intensive approach. On the other hand, the Taylor series approach can be used for non-linear models but often requires computationally intensive procedures (Pronzalo and Walter, 1997). Our proof included in Supplementary Material 1 demonstrated the mathematical model is globally structurally identifiable, meaning it is theoretically possible to design an experiment in such a way that the resulting post-calibration parameters are unique and reliable.

Once the mathematical model passes the test for structural identifiability, the quality of the experimental data for model parameter estimation should be evaluated. This test is often called the practical identifiability as it evaluates the influence of data quality on the credibility of the model parameters to be estimated. Ideally, one would design a targeted experiment to enhance the quality of data obtained for the calibration of a mathematical model at hand. There are methods for experimental design criteria development based on the Fisher information matrix (Batzel et al., 2013). In many cases, however, practical limitations such as cost, time, and even potential unethical conduct of these experiments prove prohibitive. It is often desirable to utilize previously collected data as part of clinical or animal investigations for model calibration. However, because such data are not collected for calibration purposes, their quality needs to be checked prior to the calibration of the mathematical model. It is often desirable to qualify a subset of data for reliable calibration particularly in the case of subject-specific mathematical modeling where a portion of the data is needed to define the parameters associated with each subject. This enables independent assessment of the mathematical model using the remaining data, as the ultimate test of predictive capability of the mathematical model. Furthermore, depending on the number of model parameters to be estimated and the associated optimization method, model calibration can be computationally expensive and take a long time to finish. In such circumstances, the quality of data should be evaluated before calibration commences, so that unidentifiable parameters can be either eliminated (e.g., by model reduction techniques) or fixed at appropriate values.

We leveraged SVD to gain insight on the information content of the data specifically for the calibration and eventual predictive capability assessment purposes. Our objective was to optimally split time series data of a subject into a calibration window and a validation window. The calibration window was selected so that: (i) it would provide sufficient information to maximize the identifiability of the model parameters; and (ii) the remaining validation segment would include hemorrhage and infusion inputs distinct from those included in the calibration window to allow for evaluation of the mathematical model’s predictive capability.

Utilization of SVD in this pre-calibration identifiability analysis was successful in gaining such insight on relative identifiability of parameters and selecting a suitable calibration window. SVD was applied to the regressor matrix for the entire duration of the experiment (*T* = 180 min in Figure 2). For the duration of experiment, the singular values associated with α_*v*_ were larger than α_*u*_, indicating their relative order of identifiability. This is potentially due to the experimental protocol allocating the initial 15 min of the experiment to hemorrhage only followed by 15 min of zero-input period before infusion could start at 30 min. Identifiability of *K_p_* could not be directly compared with other parameters as the original parameters involved in the SVD analysis were Kp 1+αv,Kp, and Kp 1+αu. Identifiability of *K_p_* was later confirmed as part of post calibration identifiability analysis. Based on the SVD results and that the remaining hemorrhage inputs were at 50 and 70 min, a calibration window between 0 and 50 min was selected and we proceeded to calibrate the mathematical model using data in that window.

A quadratic cost function was used for non-linear least square optimization for model calibration. This is consistent with the choice of the cost function and optimization method for most studies on the mathematical models for hemorrhage and fluid resuscitation (Bighamian et al., 2016b, 2017). However, as stated in the section “Model Calibration,” the cost function utilized in our study assumed that the experimental noise was proportional to the magnitude of the true change in BV. This assumption is supported by published literature (Aladangady et al., 2008).

The resulting calibrated parameters (Table 2) demonstrate that despite α_*u*_ being associated with non-zero singular values (i.e., the regressor matrix at 50 min having a full rank for all subjects) this parameter is identifiable at *T_*C*_* = 180 (results not shown) but may be unidentifiable at *T*_*C*_ = 50 min for most of the subjects. Moreover, for the few subjects for which α_*u*_ was identified (subjects 3, 13, and 16), α_*v*_ was unidentifiable for subject 3 and the identified value for *K_p_* took values significantly beyond the cohort mean for subjects 13 and 16. The calibration step was therefore repeated after fixing α_*u*_ to an average value of 3 based on average value of α_*u*_ identified from four Ringer’s infusion protocols reported in previous works (Bighamian et al., 2016b). Since subjects 3 and 16 continued to have implausible parameter values, they were deemed as outliers and examples of subjects for which the mathematical model fails to provide a valid BV prediction.

We suspect that the presence of a physiological mechanism currently unaccounted for in the mathematical model may have resulted in its failure in these few subjects. Closer inspection of the experimental protocol for subject 3 revealed that despite a relatively small amount of infused fluid prior to *T*_*C*_ = 50 min, the BV was restored to baseline at this time. This could be due to the activation of additional unmodeled compensatory mechanisms responsible for fluid shift resulting in higher sensitivity to fluid gain and lower sensitivity to fluid loss in the vascular compartment compared with other subjects. This in turn could have impacted the estimation of α_*v*_ as by definition this parameter is the ratio of fluid loss of intravascular and interstitial compartments. For subject 16, the high *K_p_* value could be due to an unmodeled mechanism that influences fluid shift between intravascular and interstitial compartments. In other words, a physiological mechanism affecting balance of oncotic and hydrostatic pressure such as lymphatic flow (Taylor, 1981; Huxley and Scallan, 2011) could make rate of fluid loss different than fluid gain. The Current mathematical model assumes fluid shift between the compartments without consideration of fluid shift direction, i.e., *K_p_* is the same for intravascular fluid gain and loss. As such, the model structure may not adequately estimate *K_p_* for this subject.

Once calibrated parameters are obtained, their practical identifiability should be further investigated to: (i) ensure post-calibration uniqueness; and (ii) yield uncertainty in parameter estimates. We first confirmed the identifiability of model parameters using cost function contour plots for the three-parameter model depicted in Figure 3, which confirms the previous calibration and pre-calibration identifiability results by showing unbounded contours in the direction of α_*u*_ and bounded contours for the other model parameters (i.e., α_*v*_ and *K_p_* are identifiable). We also visualized contour plots for all subjects using the two-parameter model, to verify they exhibited bounded contours (results not shown).

The next set of results involves uncertainty quantification of parameter estimates (Figure 5). The results show significant variability in both size and direction of the asymptotic 95% confidence region amongst subjects, which is expected considering the variability in subject-specific fluid infusion profiles magnified by the inherent inter- and intra-subject variabilities. Despite this variability, the identifiability results previously stated in the pre-calibration step can be verified by comparing the confidence intervals of the parameters (Table 3) or the shape of the ellipsoidal region. Note that the UQ step only involves the uncertainty in the calibrated parameters and does not embody the complete uncertainty associated with the model output because different sources of experimental error such as errors in the actual blood withdrawal rate and infusion rate were not simulated.

Propagation of uncertainty was carried out *via* Monte Carlo parameter sampling based on the uncertainty quantified in the previous step. Together with the proportionality constant of 0.2 (discussed in section “Experimental Uncertainty Quantification”) associated with the standard deviation of the experimental measurement, the 95% confidence intervals for both model prediction and experimental measurements can be compared as depicted in Figure 6. Overall, a qualitative comparison of model prediction to experiment show varying degree of predictive capability. Model predictions mostly aligned with experiments in subjects who did not reach normovolumia in the course of the therapy. There were instances of mathematical model under predicting (subjects 1, 18, and 19) and over predicting (subjects 13 and 14) the BV response. For quantitative validation and predictive capability assessment, we were interested in the segment of the time series independent of calibration, i.e., from *T*_*c*_ = 50 min to *T* = 180 min. As such, two physiologically relevant validation tests were developed at two critical times within this window.

When replacement fluid is infused to subjects under hemorrhagic shock, it is critical to avoid both under-infusion and over-infusion with the goal of maintaining BV at an ideal level which can be the subject’s normovolumic state (i.e., restoration to baseline BV). As such, it is essential for the mathematical model to be able to predict two physiologically relevant scenarios: (i) whether fluid infusion regimen will result in the achievement of normovolumic state at the conclusion of fluid therapy (*T* = 180 min); and (ii) whether normovolumia is achieved at the critical time when equal volume of fluid is infused compared to blood loss. The binary validation test results indicate that model predictions agree with the experimental results in 82% and 75% of the subjects for scenario i and ii, respectively (Table 4).

For the first scenario, the mathematical model failed in predicting volumetric state in 4 subjects (1, 13, 18, and 19). While for two subjects (1 and 19) the predictions were drastically different from experimental results, the predictions for the other two subjects (13 and 18) were very close to the observed BV and missed the binary test by a small margin. In fact, the small difference between predictions and experimental results in these two subjects may not be deemed physiologically significant.

The second scenario was evaluated in 12 subjects only, as the rest of the subjects did not have protocols to allow for infusion volume to ever equal the preset hemorrhage volume. In this group, the mathematical model failed in three subjects (8, 18, and 19). For subjects 18 and 19, the time when the volume of infusion equaled that of hemorrhage was very close to 180 min (174 and 177 min, respectively). Thus, for these subjects, this scenario does not offer new information compared to the previous test and only confirms the previous scenario results.

In this work, we have demonstrated the process of validating a subject-specific BV kinetics model for the purpose of volume status prediction and demonstrated the mathematical model’s utility for this context of use. There are, however, a number of limitations in our work both with regard to the individual steps of the process as well as the overall approach, which are discussed below.

First, we evaluated the credibility of a simple subject-specific mathematical model with few parameters. While the order of credibility assessment steps is likely to remain the same, the actual method used in each step may not be generalizable to mathematical models of varying complexity and properties (e.g., non-linearity or time-varying behavior). Second, for some animal subjects the study protocols did not allow for evaluation of model performance at the time when cumulative infusion was equal to cumulative hemorrhage. Furthermore, fluid responsiveness in our work has been defined based on the changes in BV measurements. In pre-clinical and clinical setting, measured physiologic variables such as cardiac output or blood pressure are normally used for assessment of fluid responsiveness. That said, the clinical relevance of BV measurements and potential insight they offer toward subject resuscitation management is well documented (Manzone et al., 2007; Yu et al., 2011; Ramasawmy et al., 2018). In addition, the post calibration identifiability results may change based on the optimization scheme. While we used a quadratic cost function, use of regularization methods may improve practical identifiability of some of the parameters (Tivay et al., 2020).

Lastly, the lack of experiments intended and designed for model validation also often results in absent or incomplete collection of information necessary for quantification of experimental uncertainty. In addition to the neglected accuracy specifications for instruments, data processing for such experiments often fails to account for measurement sensitivities for particular experimental parameters. Although our experimental uncertainty quantification is justified by the referenced literature which used a validated measurement technique, we acknowledge that differences in experimental conditions, subjects, and conduct could render the derived proportionality constant inaccurate. Potential mischaracterization of this value could alter the conclusions of the validation test.

## Conclusion and Future Work

We have evaluated the credibility of a subject-specific mathematical model for BV kinetics using a step-by-step workflow. Based on this study, the mathematical model is structurally identifiable. Practical identifiability with quantified parametric uncertainty can be established for two model parameters for the specific experimental protocol used for parameter estimation. The BV kinetics model demonstrated predictive capability in 82% and 75% of subjects at conclusion of fluid therapy and at the time at which the amount of infused fluid is equal to fluid loss, respectively. The broad applicability and utility of the process followed to produce these results needs to be evaluated on additional mathematical models of varying complexity. In particular, future efforts should be focused on utilizing the proposed workflow on the credibility assessment of subject-specific mathematical models for cardiac output and blood pressure responses to hemorrhage and fluid resuscitation using prospectively designed animal experiments. Furthermore, future work can be directed toward comparing the predictive performance of the BV model with an autoregressive model to derive insight on structural soundness of the BV model and quantify the value of using a mechanistic modeling approach for this application.

## Data Availability Statement

The datasets analyzed in this study can be obtained from George C. Kramer at the University of Texas Medical Branch (gkramer@utmb.edu).

## Ethics Statement

The animal study was reviewed and approved by Animal Care and Use Committee of the University of Texas Medical Branch at Galveston, with adherence to National Institutes of Health guidelines for care and use of laboratory animals (ILAR Publication, 1996 NRC ISBN 0-309-05577-3).

## Author Contributions

BP developed the validation workflow and drafted all version of the manuscript. RB developed the model that is tested in this work. CS and J-OH provided feedback and comments on the manuscript. PP provided feedback, comments, and assisted with organization of the workflow. All authors contributed to the article and approved the submitted version.

## Conflict of Interest

The authors declare that the research was conducted in the absence of any commercial or financial relationships that could be construed as a potential conflict of interest.

## Publisher’s Note

All claims expressed in this article are solely those of the authors and do not necessarily represent those of their affiliated organizations, or those of the publisher, the editors and the reviewers. Any product that may be evaluated in this article, or claim that may be made by its manufacturer, is not guaranteed or endorsed by the publisher.
