# Supplementary material for: Credibility Assessment of a Subject-Specific Mathematical Model of Blood Volume Kinetics for Prediction of Physiological Response to Hemorrhagic Shock and Fluid Resuscitation
Source: Front Physiol. 2021 Sep 16;12:705222. doi: 10.3389/fphys.2021.705222 (PMC8481867; doi:10.3389/fphys.2021.705222)
Supplement: Supplementary file 1 [file Data_Sheet_1.pdf]

Supplementary material for credibility assessment of a subject-specific mathematical model of blood volume kinetics for prediction of physiological response to hemorrhagic shock and fluid resuscitation

Bahram Parvinian, Ramin Bighamian, Christopher Scully, Jin-Oh Hahn, Pras Pathmanathan

Proof of Global Structural Identifiability

Here we prove that the three-parameter blood volume model given in Equation (1) is globally structurally identifiable. The general problem formulation for determination of global structural identifiability (GSI) or local structural identifiability (LSI) properties of a model ($\mathcal{M}$) and its set of parameters P can be described as follows

$$\mathcal{M}(\mathrm{P}) = \mathcal{M}(\widetilde{\mathrm{P}}) \Rightarrow \mathrm{P} = \widetilde{\mathrm{P}},$$

where $\tilde{P}$ is the set of parameters for the model $\mathcal{M}$ and P is the set of parameters of the modeled process. The above equation essentially implies that if model structure $\mathcal{M}$ captures the process without any error (i.e., $\mathcal{M}$ is a perfect representation of the system), then for $\mathcal{M}$ to be structurally identifiable, P needs to be unique. In the case of GSI the parameters are proven to be unique globally while for LSI, there is a neighborhood within which the uniqueness of the parameters can be guaranteed.

Consider the following general state space formulation for a linear time-invariant system:

$$\dot{\mathbf{x}}(\mathrm{t}) = \mathrm{A}\mathbf{x}(\mathrm{t}) + \mathrm{B}\mathbf{u}(\mathrm{t})$$

$$\mathbf{Y} = \mathrm{C}\mathbf{x}(\mathrm{t}) + \mathrm{D}\mathbf{u}(\mathrm{t}),$$

with initial condition $\mathbf{x}(\mathrm{t}_0) = \mathbf{x}_0$, where **x** are the state variables, **Y** are the measured quantities, and **u** are the model inputs u(t) and v(t). Converting the three parameter BV kinetic model (Equation 1) to state space form leads to the following state, input, output, and direct transmission matrices (see Bighamian *et al*, 2018 in bibliography list for additional details):

$$\dot{\mathrm{x}}(\mathrm{t}) = \begin{bmatrix} -\mathrm{K_p} & 0 & \dfrac{\mathrm{K_p}}{(1+\alpha_\mathrm{u})} & \dfrac{-\mathrm{K_p}}{(1+\alpha_\mathrm{v})} \\ -1 & 0 & \dfrac{1}{(1+\alpha_\mathrm{u})} & \dfrac{-1}{(1+\alpha_\mathrm{v})} \\ 0 & 0 & 0 & 0 \\ 0 & 0 & 0 & 0 \end{bmatrix} \mathrm{x}(\mathrm{t}) + \begin{bmatrix} 1 & -1 \\ 0 & 0 \\ 1 & 0 \\ 0 & 1 \end{bmatrix} \begin{bmatrix} \mathrm{u}(\mathrm{t}) \\ \mathrm{v}(\mathrm{t}) \end{bmatrix}, \quad \mathrm{x}(t_0) = \mathrm{x}_0$$

$$\Delta V_B = [1 \quad 0 \quad 0 \quad 0]\,\mathrm{x}(\mathrm{t})$$

where $x(t) = \left[\Delta V_B \quad \int_0^t e_B(\tau)\, d\tau \quad \int_0^t u(\tau)\, d\tau \quad \int_0^t v(\tau)\, d\tau\right]^T$ with $e_B(t) = \frac{1}{1+\alpha_u}\int_0^t u(\tau)\, d\tau - \frac{1}{1+\alpha_v}\int_0^t v(\tau)\, d\tau - \Delta V_B$

The transfer function expression of the state space BV kinetic model can be computed using the following:

$$M(s) = C(sI - A)^{-1}B + D$$

Where $A = \begin{bmatrix} -K_p & 0 & \frac{K_p}{(1+\alpha_u)} & \frac{-K_p}{(1+\alpha_v)} \\ -1 & 0 & \frac{1}{(1+\alpha_u)} & \frac{-1}{(1+\alpha_v)} \\ 0 & 0 & 0 & 0 \\ 0 & 0 & 0 & 0 \end{bmatrix}$, $B = \begin{bmatrix} 1 & -1 \\ 0 & 0 \\ 1 & 0 \\ 0 & 1 \end{bmatrix}$, $C = [1 \quad 0 \quad 0 \quad 0]$, $D = 0$

Substitution of state, input, and output matrices of the BV kinetic model with block-wise inversion leads to:

$$(sI - A)^{-1} = \begin{bmatrix} E^{-1} & \frac{-1}{s}E^{-1}F \\ 0 & \frac{1}{s}I \end{bmatrix}$$

with the following block assignments:

$$E = \begin{bmatrix} s + K_p & 0 \\ 1 & s \end{bmatrix}, F = \begin{bmatrix} \frac{-K_p}{(1+\alpha_u)} & \frac{K_p}{(1+\alpha_v)} \\ \frac{-1}{(1+\alpha_u)} & \frac{1}{(1+\alpha_v)} \end{bmatrix}.$$

Computing each block:

$$E^{-1} = \frac{1}{s(s + K_P)}\begin{bmatrix} s & 0 \\ -1 & s + K_P \end{bmatrix}$$

$$\frac{-1}{s}E^{-1}F = \frac{-1}{s^2(s + K_P)}\begin{bmatrix} s & 0 \\ -1 & s + K_P \end{bmatrix}\begin{bmatrix} \frac{-K_p}{(1+\alpha_u)} & \frac{K_p}{(1+\alpha_v)} \\ \frac{-1}{(1+\alpha_u)} & \frac{1}{(1+\alpha_v)} \end{bmatrix}$$

$$= \frac{1}{s^2(s+K_P)}\begin{bmatrix} \frac{sK_P}{(1+\alpha_u)} & -\frac{sK_P}{(1+\alpha_v)} \\ \frac{-K_P}{(1+\alpha_u)} + \frac{s+K_P}{(1+\alpha_u)} & \frac{K_P}{(1+\alpha_v)} - \frac{s+K_P}{(1+\alpha_v)} \end{bmatrix},$$

we obtain

$$(\mathrm{sI}-\mathrm{A})^{-1}=\frac{1}{\mathrm{s}(\mathrm{s}+\mathrm{K_P})}\begin{bmatrix} \mathrm{s} & 0 & \frac{\mathrm{K_p}}{(1+\alpha_\mathrm{u})} & \frac{-\mathrm{K_p}}{(1+\alpha_\mathrm{v})} \\ \vdots & \vdots & \vdots & \vdots \end{bmatrix}.$$

Therefore, the transfer function representation of $\mathcal{M}$ is

$$\mathcal{M}(\mathrm{P})=\mathrm{C}(\mathrm{sI}-\mathrm{A})^{-1}\mathrm{B}=\frac{1}{s(s+K_P)}[s+\frac{K_P}{(1+\alpha_\mathrm{u})} \qquad -\mathrm{s}-\frac{K_P}{(1+\alpha_\mathrm{v})}]$$

For $\mathcal{M}$ to be GSI we need to show:

$$\mathcal{M}(\mathrm{P})=\mathcal{M}(\widetilde{\mathrm{P}})\Rightarrow\mathrm{P}=\widetilde{\mathrm{P}}$$

Since entries of $\mathcal{M}$ are in canonical form, for the first entry we have:

$$\frac{\mathrm{s}+\left(\frac{\mathrm{K_p}}{1+\alpha_\mathrm{u}}\right)}{\mathrm{s}^2+\mathrm{sK_p}}=\frac{\mathrm{s}+\left(\frac{\widetilde{\mathrm{K_p}}}{1+\widetilde{\alpha_\mathrm{u}}}\right)}{\mathrm{s}^2+\mathrm{s}\widetilde{\mathrm{K_p}}}$$

$$=>\mathrm{K_p}=\widetilde{\mathrm{K_p}}\text{ and }\alpha_\mathrm{u}=\widetilde{\alpha_\mathrm{u}}$$

and similarly from the second entry:

$$\alpha_\mathrm{v}=\widetilde{\alpha_\mathrm{v}}$$

as required.
